# Biodegradation of caffeine by whole cells of tea-derived fungi *Aspergillus sydowii, Aspergillus niger* and optimization for caffeine degradation

**DOI:** 10.1186/s12866-018-1194-8

**Published:** 2018-06-05

**Authors:** Binxing Zhou, Cunqiang Ma, Hongzhen Wang, Tao Xia

**Affiliations:** 1 State Key Laboratory of Tea Plant Biology and Utilization, Anhui Agricultural University, Hefei, 230036 Anhui China; 2 Henan Key Laboratory of Tea Comprehensive Utilization in South Henan, Xinyang Agriculture and Forestry University, Xinyang, 464000 Henan China; 3 College of Long Run Pu-erh Tea, Yunnan Agricultural University, Kunming, 650201 Yunnan China

**Keywords:** Tea, Fungi, Biodegradation, Caffeine, Fermentation

## Abstract

**Background:**

Pu-erh tea is a traditional Chinese tea and produced by natural solid-state fermentation. Several studies show that the natural microbiota influence caffeine level in pu-erh tea. Our previous research also found that the caffeine declined significantly (*p* < 0.05) in the fermentation, which suggested that the caffeine level could be influenced by specific strains. The purpose of this study was to isolate and identify microorganisms for caffeine degradation, and this research explored the degradation products from caffeine and optimal condition for caffeine degradation.

**Results:**

11 Fungi were isolated from pu-erh tea fermentation and 7 strains could survive in caffeine solid medium. Two superior strains were identified as *Aspergillus niger* NCBT110A and *Aspergillus sydowii* NRRL250 by molecular identification. In the substrate tests with caffeine, *A. niger* NCBT110A could use caffeine as a potential carbon source while glucose is absent, *A. sydowii* NRRL250 could degrade 600 mg/L caffeine completely in a liquid medium. During the degradation product analysis of *A. sydowii* NRRL250, theophylline and 3-methlxanthine were detected, and the level of theophylline and 3-methlxanthine increased significantly (*p* < 0.05) with the degradation of caffeine. The single factor analysis showed that the optimum conditions of caffeine degradation were 1) substrate concentration of 1200 mg/L, 2) reaction temperature at 30 °C, and 3) pH of 6. In the submerged fermentation of tea infusion by *A. sydowii* NRRL250*,* 985.1 mg/L of caffeine was degraded, and 501.2 mg/L of theophylline was produced.

**Conclusions:**

Results from this research indicate that *Aspergillus sydowii* NRRL250 was an effective strain to degrade caffeine. And theophylline and 3-methlxanthine were the main caffeine degradation products.

**Electronic supplementary material:**

The online version of this article (10.1186/s12866-018-1194-8) contains supplementary material, which is available to authorized users.

## Background

Caffeine (1, 3, 7-trimethylxanthine or 3, 7-dihydro-1, 3, 7-trimethyl-1H-2, 6dione) belongs to a group of compounds known as purine alkaloids. Caffeine is a key flavor substance in many popular drinks, especially in tea and coffee. Although caffeine has lots of benefits, such as regulating the central nervous system, excess caffeine intake could develop hypertension and induce osteoporosis. Based on the recent researches, caffeine content only changes in different physiological metabolism of tea tree (*Camellia sinesis* (L.) O. Kuntze) [1], and caffeine content is influenced by tea tree varieties [2], but caffeine level remains stable among different kind of teas, which showed that tea processing cannot impact caffeine content [3, 4].

Pu-erh tea (pu-erh shucha) (PET) is produced though a natural solid-state fermentation (SSF) process with sun-dried green tea leaves (*Camellia sinensis var. assamica* (JW Masters) Kitamura) as the raw material [5–7]. PET has been produced and drank by some minorities of the Yunnan people in China for centuries [8, 9]. Microorganisms, involved in pu-erh tea solid-state fermentation (PETSSF), have been mainly studied using culture-based approaches and culture-independent approaches [6–12]. Many fungi and yeast have been isolated from PET, especially *Aspergillus niger, A. tubingensis, A. fumigatus, A. acidus, A. awamori, Penicllium sp., Rhizomucor pusillus, Rhizomucor tauricus, Blastobotrys adeninivorans, Arxula adeninivorans, Pichia farinose* and *Candida tropicalis*, which have been reported widely [8–16].

Due to the participation of microorganisms, caffeine content is changeable during PETSSF [17–20]. *Cephalosporium acremonium* dramatically increases 60–70% caffeine content during PETSSF, whereas *Saccharomycetes sp.* and *A. niger* could potentially reduce caffeine content [19–21]. In addition, the level of caffeine content is relatively stable with the effect of *A. fumigatu* and *Lactobacillus sp.* [14]. Therefore, microorganisms have a certain effect on caffeine and other purine alkaloids [22].

In this paper, samples from PETSSF were used to select target strains with the capability of caffeine degradation. This report found that *Aspergillus sydowii* NRRL250 leads caffeine biodegradation. In addition, this paper investigated the effect of *Aspergillus niger* NCBT110A on caffeine degradation.

## Methods

### Ethics statement

No specific permits were required for the described study. No specific permissions were required for these locations/activities, because specimens used in this study were manufactured in the laboratory.

### Material and reagents

Assam sun-dried green tea leaves (*C. sinensis* var. *assamica* (JW Masters) Kitamura) with moisture content 6.25% by weight were obtained from Yunnan province, China. Caffeine (purity about 95%), used in culture medium, was purchased from Tianjin Guangfu fine chemical industry institute, China. Caffeine (≥99%), theophylline (≥99%) and 3-methylxanthine (≥99%) standards were purchased form Sigma Company, USA. SP fungal DNA kit, DNA marker, polymerase chain reaction (PCR) spread reagent, internal transcribed spacers (ITS): ITS1 (5`-TCCGTAGGTGAACCTGCGG-3`) and ITS4 (5`-TCCTCCGCTTATTGATAGC-3`) were purchased from TaKaRa Company, Japan. High performance liquid chromatography (HPLC) grade of acetonitrile was purchased from Beijing Mreda Biotechnology Company, China. Other reagents were of analytical grade.

### A solid-state fermentation

In this study, PTSSF was manufactured in Tea Processing Laboratory, College of Long Run Pu-erh Tea, Yunnan Agricultural University, Kunming of Yunnan province to simulate pu-erh tea industrial production. Sun-dried green leaves (400 g) were moistened with distilled water (220 mL) to achieve a final moisture content of 35% (*w*/w) [23]. SSF was carried out with the natural microbiota exist on the leaves [21]. The whole fermentation process continued for 35 d in a nature condition. The leaves were turned over with sprayed by moderate sterile water for every 5 days to ensure a homogeneous fermentation. Samples were collected periodically from fermentation for chemical and microbial analyses [23]. In addition, parallel tests were carried out to ensure the data reliability.

### Isolation and identification of target strains

Fermented sample would be used to isolate the fungi and they were counted by dilution plating method [23]. The colony forming units (CFU) was calculated by per gram dry weight of tea leaves after 2 days of cultivation at 30 °C. The caffeine content of related samples was determined by HPLC [24].

The target strains were inoculated and cultivated in the potato dextrose agar (PDA) and Czapek-Dox mediums at 30 °C, respectively. The colony morphological characteristics and conidia structure were observed after cultivation for 5 d. The target strains grew aerobically as pure cultures in 20 mL of Czapek-Dox liquid medium in 125 mL shake flasks at 30 °C, 250 rpm, for 2 d. The fresh cells were obtained by centrifugation at 1700 g for 5 min and freeze-dried at − 80 °C [10, 23]. DNA was extracted by using SP fungal DNA kit. The extracted DNA was subject to amplify the ITS region, the universal fungal primers ITS1 and ITS4 were used in the PCR [11, 12, 23]. The final volume of 50 μL, 1.0 *μ*L of containing template DNA, 5 *μ*L of 10 x buffer, 5 *μ*L of dNTPs (2.5 mM), 0.5 *μ*L of Taq polymerase, 1.0 *μ*L (10 μM) of each primer, and 36.5 *μ*L of sterile distilled water were used to implement amplifications [12, 23]. The PCR reaction procedure was as follows. Pre-degeneration at 95 °C for 5 min, degeneration at 94 °C for 1 min, annealing at 54 °C for 1 min, extension at 72 °C for 1.5 min, with 35 cycles, extension at 72 °C for 10 min [15]. It was stored at 10 °C in the end of the reaction process.

The PCR mixtures were analyzed by using ABI3730 automatic DNA sequencer (Applied Biosystems, USA). The received sequence was sent to Genbank of NCBI to seek semi-root sequence. ITS1–5.8S rRNA-ITS2 gene sequence of related strains were transferred and compared with ClustalX 1.8. The evolution distance was calculated through a Kimura2-parameter of the MEGA 4 Soft. Neighbor-Joining method was used to establish phylogenetic trees. 1000 random samples were taken to calculate Bootstrap for evaluation of the phylogenetic confidence level.

### Growth of tea-derived fungi in a solid medium

Qualitative screenings were carried out on Petri dishes containing a solid culture medium with glucose (2% *w*/*v*) (control culture) and a selection medium with caffeine instead of glucose in three different concentrations: 600, 1200 and 1800 mg/L per plate, respectively [25]. Fungal mycelia from recent cultures were transferred to the surface of the agar plates with an inoculating loop. Strains were incubated at 30 °C for 5 d. Compared with the control culture, the strains utilized caffeine was estimated by the size of the colony grown on the plates (Table 1).

**Table 1:** Growth of tea-derived fungi in agar solid medium (2% *w*/*v*) with glucose (2% w/v) (control culture) or presence of caffeine instead of glucose (30 °C, 5 d, pH 7.0)

Tea-derived fungi	Strains growth (cm)			
	Control culture	600 mg/L of caffeine	1200 mg/L of caffeine	1800 mg/L of caffeine
No. 1	3.5 × 3.5	1.0 × 1.0	2.0 × 1.5	2.0 × 2.0
No. 2	4.0 × 2.5	0.5 × 0.5	1.0 × 0.5	1.0 × 0.5
No. 3	3.0 × 2.5	1.0 × 0.5	1.0 × 1.0	1.0 × 1.0
No. 4	3.0 × 3.0	no growth	0.5 × 0.5	1.0 × 0.5
No. 5	4.0 × 2.5	3.0 × 2.5	2.5 × 2.5	3.0 × 2.5
No. 6	3.0 × 2.5	0.5 × 0.5	1.0 × 0.5	1.0 × 0.5
No. 7	3.0 × 3.0	no growth	0.5 × 0.5	1.0 × 1.0

### Growth of tea-derived fungi in a liquid medium

Spore solutions of target strains were prepared by growing the fungi for 5 d at 30 °C in dishes containing solid culture medium with glucose [25]. Two loopfuls of target strains were transferred aseptically from a dish slant into 25 mL of a sterile liquid medium (per liter: potato starch 4 g, dextrose 20 g, chloramphenicol 0.1 g) with 600 mg/L of caffeine in a 125 mL Erlenmeyer flask. The flask was incubated aerobically on an incubator shaker (250 rpm) at 30 °C for 48 h. The volume of the seed was 10% (*v*/v) of total initial volume [23]. The flask was incubated in an orbital shaker during 3, 6, 9, 12 and 15 d (130 rpm, 30 °C). The mycelium was collected after the culture was filtered in a Buchner funnel, and rinsed in 20 mL of water: ethyl acetate (1:1) [25]. The mycelial mass was determined as fungal dry mass after drying at 35 °C for 24 h [25]. Biodegraded products of caffeine were analyzed by HPLC [24]. The results were summarized in Table 2.

**Table 2:** Quantitative biodegradation of caffeine by *A. sydowii* and *A. niger* on 5, 10 and 15th day

Reaction time (d)	Fungal dry mass (g)	*C*^a^_caffeine_ (mg/L)	*C*^a^_theophylline_ (mg/L)	% of caffeine degraded ^b^
*A. sydowii* NRRL250 (600 mg/L of caffeine)				
5	0.23 ± 0.02	431.5 ± 39.7	40.4 ± 1.0	28.1 ± 6.6
10	0.24 ± 0.02	134.8 ± 6.5	209.9 ± 22.6	77.5 ± 1.1
15	0.22 ± 0.01	3.7 ± 0.8	262.6 ± 20.7	99.4 ± 0.1
*A. niger* NCBT110A (600 mg/L of caffeine)				
5	0.28 ± 0.03	592.6 ± 3.1	NF	1.2 ± 0.5
10	0.29 ± 0.02	580.0 ± 2.9	NF	3.3 ± 0.5
15	0.27 ± 0.01	577.3 ± 6.0	NF	3.8 ± 1.0

^a^*C* Concentration determined by HPLC

^b^% of caffeine degraded was estimated as follow: of caffeine degraded = (C_0_-C_t_)/C_0_*100% (1)

In Eq. (1) C_0_ was the initial caffeine concentration (mg/L), C_t_ was the final caffeine concentration (mg/L) after the fermentation

All data are presented as mean ± SD. *NF* not found

### Biodegradation in a liquid medium

Studies were performed during 15 d in the liquid medium to evaluate the kinetic parameters for biodegradation reactions of caffeine. Effects of substrate concentration, reaction temperature and pH on the kinetic parameters were investigated [25].

1. Substrate concentration. The seed was inoculated into the sterile liquid medium by 10% (*v*/v) of above noted inoculum with different initial caffeine concentration (600, 1200 and 1800 mg/L, respectively). The flask was incubated in an orbital shaker during 5, 10 and 15 d (130 rpm, 30 °C).
2. Reaction temperature. The seed was inoculated into the sterile liquid medium by 10% (*v*/v) of above noted inoculum with the initial caffeine concentration of 1200 mg/L. The flask was incubated in an orbital shaker with different reaction temperature (25, 30, 35 °C, respectively) during 5, 10 and 15 d.
3. pH. The seed and liquid medium were adjusted for different pH (5.0, 6.0,7.0, respectively) by phosphate buffer. The seed was inoculated into the sterile liquid medium by 10% (v/v) of above noted inoculum with the initial caffeine concentration of 1200 mg/L. The flask was incubated in an orbital shaker during 5, 10 and 15 d (130 rpm, 30 °C).

Fungal dry mass was determined. The final caffeine and biodegraded products viz. theophylline and 3-methylxanthine were determined by HPLC [24].

### A submerged fermentation (SMF) of tea infusion

Sun-dried green tea leaves (1.0 g) were infused for 15 min in boiling distilled water (30 mL) and the tea infusion was made up to 30 mL with distilled water after filtration [23]. Caffeine and other functional ingredients (tea polyphenols and theabrownins) are relatively stable in high temperature condition. Based on the investigation of several thermal treatment methods (Additional file 1: Table S1), including the control (no further treatment), high temperature sterilization at 121 °C for 5 min, pasteurization at various conditions (65 °C, 30 min; 75 °C, 30 min and 80 °C, 30 min) and microwave heating (640 W, 2 min) [23], sterilization can kill viable microorganisms with minimal damage in main functional components. Therefore, sterilization was selected as the reasonable treatment for SMF.

Two loopfuls of target strains were transferred aseptically from a dish slant into 25 mL of sterile tea infusion in a 125 mL Erlenmeyer flask [23]. The flask was incubated aerobically at 30 °C for 48 h on an incubator shaker (250 rpm). The volume of the seed was 10% (*v*/v) of total initial volume of the inoculation [23]. Non-inoculation (control) and non-sterilization (natural treatment) were carried out. The flask was incubated in an orbital shaker for 3, 6, 9, 12 and 15 d (130 rpm, 30 °C), respectively. Fungal dry mass, caffeine and theophylline contents were determined.

## Results

### Caffeine content and fungi count during PETSSF

**Fig. 1:**
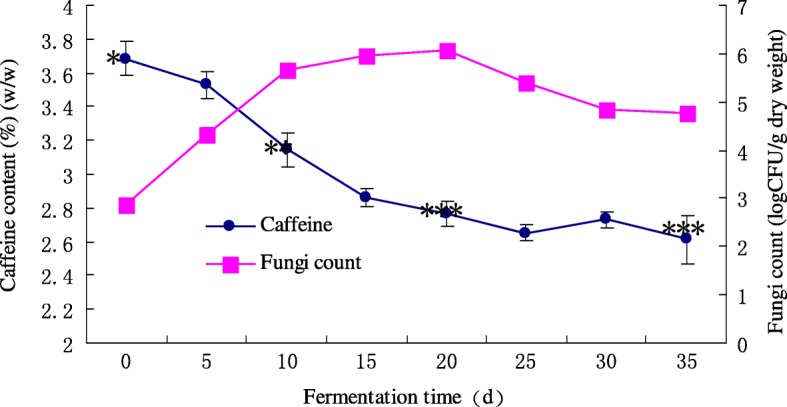
Changes in caffeine content and the fungal count of tea leaves during SSF. Data are presented as mean values ± SD. *,** and *** show the significant difference levels (*p* < 0.05) during the fermentation

The Fig. 1 shows that the fugal colony count dramatically increased from 0 to 10 days. Then, it increased slowly from 4.8 × 10^5^ CFU/g dry weight of tea leaves to 1.2 × 10^6^ CFU/g on day 20. Due to nutrient limitation in the tea leaves, the colony count decreased after day 20. With changing of fungi count, caffeine content (Fig. 1) declined significantly (*p* < 0.05) from 3.685 ± 0.1006% (*w*/w) to 2.612 ± 0.1398% (w/w) during the fermentation. According to the analysis above, it suggested that the fungal colonies cause the decrease of caffeine content. Through separation and purification, 11 fungi were isolated from PETSSF.

### Screening result of tea-derived fungi in a solid medium

The screening was carried out in an agar solid medium for selecting the tea-derived fungi able to utilize caffeine. In order to evaluate the biocatalytic potential for degradation of caffeine, all investigated strains were inoculated into an agar solid medium with presence of glucose and they were also inoculated into a set of agar solid medium with presence of different concentration caffeine. The sizes of the colonies were measured and showed in Table 1.

**Fig. 2:**
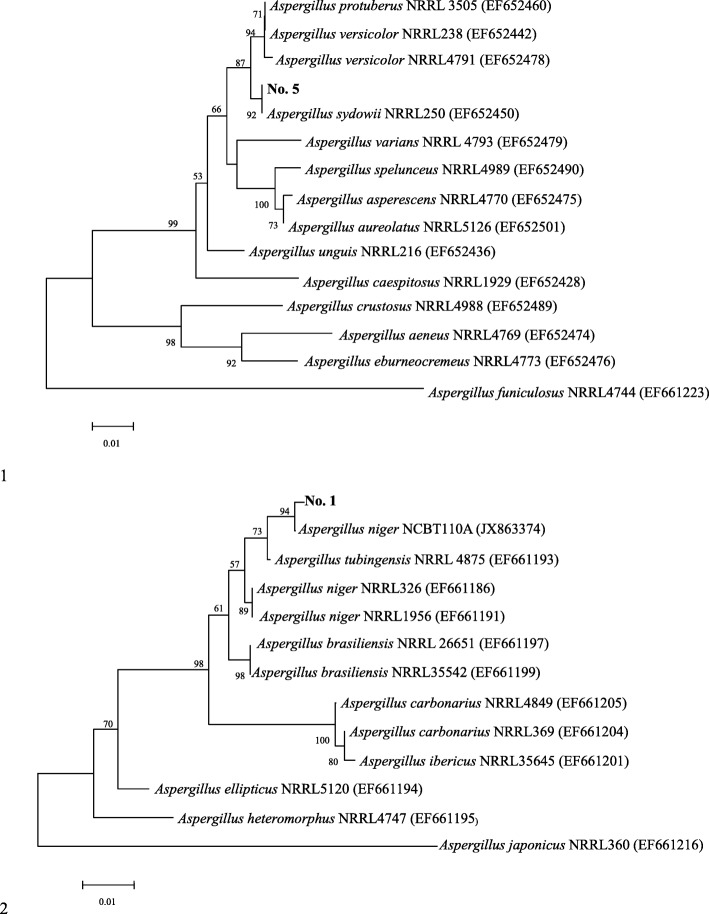
Phylogenetic tree of the target strains (strains No. 5 and No.1)

Among 11 tea-derived fungi, 7 strains could survive in the agar solid medium (2% *w*/*v*) with caffeine alone. Strains No. 1, No. 3 and No. 5 showed the best growth in all concentrations evaluated. And, stains No. 4 and No. 7 had no growth in low caffeine concentration, which showed that the utilization ratio of caffeine was restricted. If fungi had a higher growth in a low caffeine concentration, it may indicate that fungi could use caffeine as a carbon source directly or indirectly. Because strains No. 5 and No. 1 had a high growth rate at the lowest caffeine concentration, they were considered as the potential target strains. Colony characteristics and microscopic structure of strain No. 5 were showed in Additional file 2: Figure S1 and S2, colony characteristics and microscopic structure of strain No. 1 were showed in Additional file 2: Figure S3 and S4. Through molecular identification (Additional file 3: Figure S5), strain No. 5 was identified as *Aspergillus sydowii* NRRL250 with 99.8% homology, strain No. 1 was identified as *Aspergillus niger* NCBT110A with 99.8% homology (Fig. 2).

### Biodegradation of caffeine by *A. sydowii* NRRL250 and *A. niger* NCBT110A

For the biodegradation reaction in a liquid medium, the selected strains (*A. sydowii* NRRL250 and *A. niger* NCBT110A) were inoculated into the nutrient medium with the presence of caffeine. During the fermentation, fungal dry mass and caffeine content were determined. In addition, the caffeine degradation rates were calculated by the Eq. (1) to investigate the degrading capability of selected strains. For *A. niger* NCBT110A, caffeine was no significantly degraded with about 1.2, 3.3, 3.8% of caffeine degraded and theophylline was not detected in the fermentation. Caffeine degradation capability of *A. niger* NCBT110A was limited in the liquid medium, which showed that *A. niger* NCBT110A could use caffeine as a potential carbon source when glucose and other nutrients were limited or absent. For *A. sydowii* NRRL250, caffeine was almost completely degraded at 600 mg/L of caffeine (Table 2). In 15 d, caffeine had been degraded completely. As the degradation product, theophylline was determined by HPLC and increased observably from 40.4 ± 1.0 mg/L on 5 d to 262.6 ± 20.7 mg/L on 15 d. As *A. sydowii* NRRL250 has ability to degrade caffeine, it will be used to conduct the biodegradation products analysis of caffeine in below.

### Biodegradation products analysis of caffeine by *A. sydowii* NRRL250

*A. sydowii* NRRL250 was inoculated into a liquid medium with 1200 mg/L of caffeine for analysis of its biodegraded products. And caffeine, theophylline and 3-methylxanthine were determined by HPLC during 3, 6, 9, 12 and 15 d in the fermentation. Caffeine, theophylline and 3-methylxanthine contents as well as caffeine degradation rates were showed in Table 3. Degradation of approximately 7.1, 33.0, 52.8, 68.7 and 90.1% were observed in 3, 6, 9, 12 and 15 d, respectively. Both theophylline and 3-methylxanthine were detected in the fermentation, which showed that theophylline and 3-methylxanthine were the main degradation products from caffeine. Theophylline was first detected on day 3 and it increased obviously with the caffeine degradation, which showed that theophylline was a direct degradation product from caffeine through demethylation. 3-Methylxanthine was not detected on day 3 and first detected on day 6, which indicated that 3-methylxanthine might be a direct degradation product from theophylline instead of caffeine. As the secondary product, 3-methylxanthine content was far below theophylline and only 178.7 ± 10.8 mg/L was produced in 15 d.

**Table 3:** Biodegradation products analysis of caffeine by *A. sydowii* NRRL250 in a liquid medium with 1200 mg/L of caffeine

Reaction time (d)	*C*^a^_caffeine_ (mg/L)	*C*^a^_theophylline_ (mg/L)	*C*^a^_3-methylxanthine_ (mg/L)	% of caffeine degraded
3	994.9 ± 35.2	62.8 ± 11.3	NF	7.1 ± 2.9
6	804.1 ± 26.5	201.2 ± 8.4	28.4 ± 1.5	33.0 ± 2.2
9	566.3 ± 16.5	274.7 ± 14.7	38.4 ± 3.4	52.8 ± 1.4
12	375.3 ± 15.3	426.3 ± 20.8	68.1 ± 6.9	68.7 ± 1.3
15	105.0 ± 16.9	549.4 ± 29.3	178.7 ± 10.8	90.1 ± 3.0

^a^*C* Concentration determined by HPLC

All data are presented as mean ± SD. *NF* not found

### Effects of substrate concentration, reaction temperature and pH on the kinetic parameters for caffeine degradation by *A. sydowii* NRRL250

Microorganism metabolism and degradation capability were influenced by substrate concentration, reaction temperature and pH. In this paper, effects of substrate concentration, reaction temperature and pH on fungal dry mass and kinetic parameters of caffeine degradation by *A. sydowii* NRRL250 were investigated (Tables 4, 5 and 6, respectively). *A. sydowii* NRRL250 was inoculated into a liquid medium with increasing caffeine concentrations (600, 1200 and 1800 mg/L, respectively), and the flasks were incubated in an orbital shaker for 15 d (130 rpm, 30 °C). Fungal dry mass and the kinetic parameters, including final caffeine concentration (C_caffeine,f_), final theophylline concentration (*C*_theophylline,f_), final 3-methylxanthine concentration (*C*_3-methylxanthine,f_), the volumetric rate of caffeine degradation (Q_caffeine_), the volumetric rate of theophylline production (Q_theophylline_), the yield of theophylline (Y_theophylline/caffeine_) and caffeine degradation rate (% of caffeine degraded) in different substrate concentrations were showed in Table 4. There was no significant difference in fungal dry mass (*p* > 0.05). The final concentrations of theophylline and 3-methylaxthine increased significantly (*p* < 0.05), caffeine degradation rate decreased significantly (p < 0.05) in higher initial caffeine concentrations. Only about 62.9% of caffeine was degraded in 1800 mg/L caffeine concentration. By comparing the results, 1200 mg/L was an appropriate substrate concentration with the high caffeine degradation rate and the high theophylline production.

**Table 4:** Comparison of the kinetic parameters for caffeine degradation in different substrate concentrations (30 °C, 15 d, pH 7.0)^a^

Substrate concentration (mg/L)	Fungal dry mass (g)	C_caffeine,f_ (mg/L)	*C*_theophylline,f_ (mg/L)	*C*_3-methylxanthine,f_ (mg/L)	Q_caffeine_ (mg/L d)	Q_theophylline_ (mg/L d)	Y_theophylline/caffeine_	% of caffeine degraded
600	0.22 ± 0.02^A^	3.7 ± 0.8^A^	262.6 ± 20.7^A^	115.8 ± 10.1^A^	39.8 ± 0.5^A^	17.5 ± 1.4^A^	0.44 ± 0.04^A^	99.3 ± 0.1^A^
1200	0.22 ± 0.03^A^	105.0 ± 16.9^B^	549.4 ± 29.3^B^	178.7 ± 10.8^B^	73.0 ± 1.1^B^	36.6 ± 2.0^B^	0.50 ± 0.03^B^	91.3 ± 1.4^B^
1800	0.23 ± 0.02^A^	668.2 ± 37.3^C^	643.8 ± 25.3^C^	191.2 ± 4.5^B^	75.5 ± 2.5^B^	42.9 ± 1.7^C^	0.57 ± 0.01^C^	62.9 ± 2.1^C^

^a^All kinetic parameters were calculated according to Sirisansaneeyakul and others (2013) [34]

All data are presented as mean ± SD, ^A-C^*p* < 0.05 in the same column

Concentrations of caffeine, theophylline and 3-methylxanthine determined by HPLC

*C*_*caffeine,f*_ the final caffeine concentration (mg/L), *C*_*theophylline,f*_ the final theophylline concentration (mg/L), *C*_*3-methylxanthine,f*_ the final 3-methylxanthine concentration (mg/L), *Q*_*caffeine*_ the volumetric rate of caffeine degradation (mg/L d), *Q*_*theophylline*_ the volumetric rate of theophylline production (mg/L d), *Y*_*theophylline/caffeine*_ theophyline yield on caffeine (mg/mg)

**Table 5:** Comparison of the kinetic parameters for caffeine degradation on different reaction temperatures (1200 mg/L of caffeine,15d. pH 7.0)^a^

Reaction temperature (°C)	Fungal dry mass (g)	C_caffeine,f_ (mg/L)	*C*_theophylline,f_ (mg/L)	*C*_3-methylxanthine,f_ (mg/L)	Q_caffeine_ (mg/L d)	Q_theophylline_ (mg/L d)	Y_theophylline/caffeine_	% of caffeine degraded
25	0.22 ± 0.02^A^	121.6 ± 14.4^A^	478.8 ± 20.2^A^	196.6 ± 7.5^A^	71.9 ± 1.0^B^	31.9 ± 1.3^A^	0.44 ± 0.02^A^	89.9 ± 1.2^B^
30	0.22 ± 0.03^A^	105.0 ± 16.9^A^	549.4 ± 29.3^B^	178.7 ± 10.8^B^	73.0 ± 1.1^B^	36.6 ± 2.0^B^	0.50 ± 0.03^B^	90.5 ± 1.4^B^
35	0.19 ± 0.02^A^	202.0 ± 15.7^B^	618.4 ± 18.8^C^	149.8 ± 13.2^B^	66.5 ± 1.0^A^	421.2 ± 1.3^C^	0.61 ± 0.02^C^	83.2 ± 1.3^A^

^a^All kinetic parameters were calculated according to Sirisansaneeyakul and others (2013) [34]

All data are presented as mean ± SD, ^A-C^*p* < 0.05 in the same column

Concentrations of caffeine, theophylline and 3-methylxanthine determined by HPLC

*Ccaffeine,f* the final caffeine concentration (mg/L), *Ctheophylline,f* the final theophylline concentration (mg/L), *C3-methylxanthine,f* the final 3-methylxanthine concentration (mg/L), *Qcaffeine* the volumetric rate of caffeine degradation (mg/L d), *Qtheophylline* the volumetric rate of theophylline production (mg/L d), *Ytheophylline/caffeine* theophyline yield on caffeine (mg/mg)

**Table 6:** Comparison of the kinetic parameters for caffeine degradation in different pH (1200 mg/L of caffeine, 30 °C,15d.)^a^

pH	Fungal dry mass (g)	C_caffeine,f_ (mg/L)	*C*_theophylline,f_ (mg/L)	*C*_3-methylxanthine,f_ (mg/L)	Q_caffeine_ (mg/L d)	Q_theophylline_ (mg/L d)	Y_theophylline/caffeine_	% of caffeine degraded
5	0.14 ± 0.01^A^	508.5 ± 45.4^C^	245.3 ± 17.5^A^	87.8 ± 12.5^A^	46.1 ± 3.0^A^	16.4 ± 1.2^A^	0.35 ± 0.01^A^	57.6 ± 3.8^A^
6	0.22 ± 0.02^B^	41.7 ± 5.9^A^	776.5 ± 35.8^C^	125.1 ± 10.9^B^	77.2 ± 0.4^C^	51.8 ± 2.4^C^	0.67 ± 0.03^C^	96.5 ± 0.5^C^
7	0.22 ± 0.03^B^	105.0 ± 16.9^B^	549.4 ± 29.3^B^	178.7 ± 10.8^C^	73.0 ± 1.1^B^	36.6 ± 2.0^B^	0.50 ± 0.03^B^	91.3 ± 1.4^A^

^a^All kinetic parameters were calculated according to Sirisansaneeyakul and others (2013) [34]

All data are presented as mean ± SD, ^A-C^*p* < 0.05 in the same column

Concentrations of caffeine, theophylline and 3-methylxanthine determined by HPLC

*C*_*caffeine,f*_ the final caffeine concentration (mg/L), C_theophylline,f_ the final theophylline concentration (mg/L), C_3-methylxanthine,f_ the final 3-methylxanthine concentration (mg/L); *Q*_*caffeine*_ the volumetric rate of caffeine degradation (mg/L d), *Q*_*theophylline*_ the volumetric rate of theophylline production (mg/L d), *Y*_*theophylline/caffeine*_ theophyline yield on caffeine (mg/mg)

In order to compare the kinetic parameters in different reaction temperatures, *A. sydowii* NRRL250 was inoculated into an identical liquid medium with the initial caffeine concentration of 1200 mg/L, and the flasks were incubated in an orbital shaker with different reaction temperatures (25, 30 and 35 °C, respectively) for 15 d. The fungal dry mass and the kinetic parameters were showed in Table 5. In the temperature range between 25 and 30 °C, there were no significant differences in fungal dry mass, the final caffeine concentration, the volumetric rate of caffeine degradation, and caffeine degradation rate (*p* > 0.05). In 35 °C, there was no significant decline (*p* > 0.05) in fungal mass. And the final caffeine concentration, the volumetric rate of caffeine degradation and caffeine degradation rate declined significantly (*p* < 0.05). However, the theophylline concentration, the volumetric rate of theophylline production and the yield of theophylline increased significantly (*p* < 0.05) in 35 °C. The optimal temperature for caffeine degradation was 30 °C. And higher temperature promotes theophylline production.

In order to compare the kinetic parameters in different pH, phosphate buffer was used to adjust the pH of liquid medium. The fungal mass and the kinetic parameters were showed in Table 6. pH had remarkable effects on fugal dry mass and the kinetic parameters. In pH of 5, the growth and caffeine catabolism of *A. sydowii* NRRL250 were restricted. Through comparisons, pH of 6 was the optimum pH for caffeine degradation with the highest fugal dry mass, caffeine degradation rate and theophylline production.

### Applications of *A. sydowii* NRRL250 and *A. niger* NCBT110A in SMF of tea infusion

**Fig. 3:**
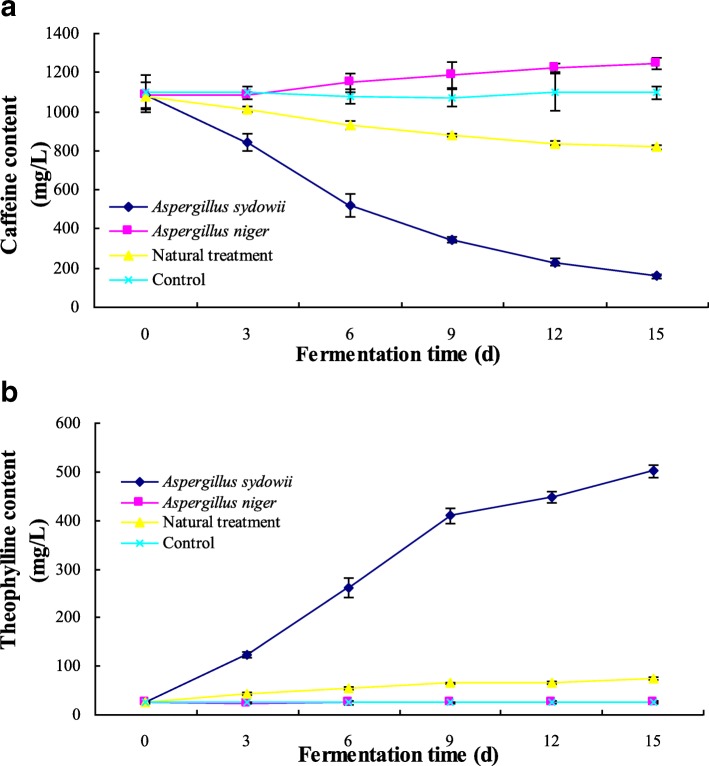
Changes in concentrations of caffeine (**a**) and theophylline (**b**) in shake flask fermentation with various pure culture. Data are presented as mean ± SD. Control was no inoculation treatment

Due to the caffeine degradation characteristic, *A. sydowii* NRRL250 was suitable to produce decaffeinated and high-theophylline tea through an inoculated fermentation. In this paper, *A. sydowii* NRRL250 was inoculated into the sterile tea infusion for SMF, caffeine and theophylline contents were determined by HPLC during 0, 3, 6, 9, 12 and 15 d. The final fungal dry mass was also determined. In addition, the SMF inoculated by *A. niger* NCBT110A, natural treatment and sterile treatment (control) were carried out to explore the influence of microorganism on caffeine. Changes of caffeine and theophylline contents were showed in Fig. 3. Fungal dry mass and the kinetic parameters of caffeine degradation were showed in Table 7.

**Table 7:** Comparison of the kinetic parameters for caffeine degradation in tea infusion fermentation (30 °C, 15d, natural pH)^a^

Strains or treatments	Fungal dry mass (g)	C_caffeine,0_ (mg/L)	C_caffeine,f_ (mg/L)	*C*_theophylline,f_ (mg/L)	Q_caffeine_ (mg/L d)	Q_theophylline_ (mg/L d)	Y_theophylline/caffeine_	% of caffeine degraded
*A. sydowii* NRRL250	0.19 ± 0.02^A^	1082.9 ± 65.8^A^	157.8 ± 10.2^A^	501.2 ± 13.5^C^	61.7 ± 5.0^B^	31.8 ± 0.8^B^	0.52 ± 0.05^B^	85.4 ± 1.7^B^
*A. niger* NCBT110A	0.24 ± 0.01^B^	1085.3 ± 64.8^A^	1248.1 ± 30.5^D^	27.2 ± 0.8^A^	ND	ND	ND	ND
Natural treatment	0.20 ± 0.02^A^	1073.9 ± 78.6^A^	817.6 ± 8.6^B^	74.7 ± 3.3^B^	17.1 ± 5.14^A^	3.3 ± 0.1^A^	0.21 ± 0.06^A^	23.6 ± 5.3^A^
Control	ND	1101.6 ± 89.5^A^	1096.4 ± 33.2^C^	25.0 ± 2.1^A^	ND	ND	ND	ND

^a^All kinetic parameters were calculated according to Sirisansaneeyakul and others (2013) [34]

All data are presented as mean ± SD, ^A-D^*p* < 0.05 in the same column

Concentrations of caffeine, theophylline and 3-methylxanthine determined by HPLC

*C*_*caffeine,0*_ initial caffeine concentration (mg/L), *C*_*caffeine,f*_ the final caffeine concentration (mg/L), *C*_theophylline,f_ the final theophylline concentration (mg/L) *Q*_*caffeine*_ the volumetric rate of caffeine degradation (mg/L d), *Q*_*theophylline*_ the volumetric rate of theophylline production (mg/L d), *Y*_*theophylline/caffeine*_ theophyline yield on caffeine (mg/mg), *ND* not determined

There were no significant changes of caffeine and theophylline contents in sterile treatment (control) (*p* > 0.05). During SMF by *A. niger* NCBT110A, caffeine increased significantly (*p* < 0.05), which showed that the caffeine degradation capability of *A. niger* NCBT110A was limited in the presence of carbohydrate and other nutriment. During SMF by *A. sydowii* NRRL250, most of caffeine (985.1 mg/L) was degraded (Fig. 3a, Table 7). As a main degradation product, theophylline increased sharply from 24.7 mg/L to 501.2 mg/L in the SMF by *A. sydowii* NRRL250 (Fig. 3b, Table 7). Because the existence of *A. sydowii* NRRL250, in natural treatment, caffeine decreased significantly with about 256.3 mg/L of caffeine degraded and theophylline increased significantly with 74.7 mg/L in 15 d (*p* < 0.05) (Fig. 3). Therefore, *A. sydowii* NRRL250 was appropriate for the production of decaffeinated tea or high-theophylline tea through an inoculated fermentation.

## Discussion

Although several effective strains were selected from the soil of tea and coffee gardens to degrade caffeine [26–28], the functional strain selected from tea was not reported. In this paper, 11 fungi were isolated from PETSSF, and 7 strains could survive in a solid medium with caffeine alone. But only 2 strains had a high growth rate at the lowest caffeine concentration, which suggested that those 2 strains used caffeine as a carbon source directly or indirectly. The two superior strains were identified as *A. niger* NCBT110A and *A. sydowii* NRRL250 by molecular identification method.

The substrate tests in the liquid medium with caffeine found that the caffeine degradation capability of *A. niger* NCBT110A was limited in the presence of glucose and other nutrients. *A. niger* NCBT110A could use caffeine as a potential carbon source when the absence of glucose. *A. sydowii* NRRL250 could degrade caffeine completely in a liquid medium with 600 mg/L of caffeine. Therefore, *A. sydowii* NRRL250 was a potentially effective strain to degrade caffeine.

In the perspective of physiology of tea tree (*C. sinesis* (L.) O. Kuntze), caffeine is synthesized in the root. Theobromine (3, 7-dimethyxanthine) is a direct precursor of caffeine anabolism and a major rate-limiting step in caffeine synthesis [29]. Theophylline (1, 3-dimethyxanthine) and 3-methylxanthine are the main degradation products in caffeine catabolism [30]. In addition, theophylline is a rate-limiting step of caffeine catabolism in the physiology of tea tree (*C. sinesis* (L.) O. Kuntze) and coffee tree (*Coffea arabica* L.). And demethylase is an important enzyme which catalyzes the reaction from caffeine to theophylline. In microbial secondary metabolites, the degradation products and degradation pathways of caffeine were not completely clear. In the substrate tests with caffeine of *A. sydowii* NRRL250, theophylline and 3-methlxanthine were detected. And theophylline and 3-methlxanthine increased significantly (*p* < 0.05) with the degradation of caffeine. Caffeine catabolism in secondary metabolites of *A. sydowii* NRRL250 was similar to the metabolites in the physiology of tea tree (*C. sinesis* (L.) O. Kuntze), theophylline and 3-methlxanthine were the main degradation products from caffeine by demethylation.

In this study, the optimum substrate concentration, reaction temperature and pH of *A. sydowii* NRRL250 were investigated. The optimum conditions of caffeine degradation were 1) substrate concentration of 1200 mg/L, 2) reaction temperature at 30 °C, and 3) pH of 6. The optimum conditions provided the relevant information for the application of *A. sydowii* NRRL250 in caffeine degradation.

In previous researches, *A. sydowii* is an important industrial and medical microorganism, which could produce monosaccharide and indole alkaloids [31–33]. In addition, *A. sydowii* could be used in biodegradation of methyl parathion [25]. Due to the caffeine degradation characteristic, *A. sydowii* NRRL250 would be applied in the production of decaffeinated tea or high-theophylline tea. In SMF, 985.1 mg/L of caffeine was degraded, and 501.2 mg/L of theophylline was produced in 15 d. Further research could be conducted in related to the caffeine degradation pathway and productive technology of decaffeinated tea by *A. sydowii* NRRL250.

## Conclusions

The purpose of this research was to screen and identify the strains which able to degrade caffeine during the PET fermentation process. The results of the research show that strain *Aspergillus sydowii* NRRL250 and strain *A. niger* NCBT110A could use caffeine as a potential carbon source when glucose and other nutrients were limited or absent. *A. sydowii* NRRL250 was an effective strain to degrade caffeine, which could be applied in the production of decaffeinated or high-theophylline tea. In addition, theophylline and 3-methlxanthine were the main degradation products from caffeine in secondary metabolites of *A. sydowii* NRRL250.

## Additional files

Additional file 1: **Table S1.** Heating method effects on microbial count and main chemical components of tea infusion. Note: All date are presented as mean ± SD, ^A-B^*p* < 0.05 in the same column, ND: not detectable, TPs is the abbreviation of tea polyphenols. (DOCX 15 kb)

Additional file 2: **Figure S1.** Colony characteristics of strain No. 5 on culture medium. **Figure S2.** Conidia structure of strain No.5 under optical microscope. **Figure S3.** Colony characteristics of strain No.1 on culture medium. **Figure S4.** Conidia structure of strain No.1 under optical microscope. (DOCX 3679 kb)

Additional file 3: **Figure S5.** ITS sequences data of the target strains. (DOCX 16 kb)

## Abbreviations

CFU: Colony forming units
DNA: Deoxyribonucleic acid
HPLC: High performance liquid chromatography
ITS: Internal transcribed spacer
MEGA: Molecular evolutionary genetics analysis
NCBI: National Center for Biotechnology Information
PCR: Polymerase chain reaction
PDA: Potato dextrose agar
PET: Pu-erh tea
PETSSF: Pu-erh tea solid-state fermentation
RNA: Ribonucleic acid
rRNA: Ribosomal ribonucleic acid
SMF: Submerged fermentation
SSF: Solid-state fermentation
