# Supplementary material for: Biodegradation of caffeine by whole cells of tea-derived fungi Aspergillus sydowii, Aspergillus niger and optimization for caffeine degradation
Source: BMC Microbiol. 2018 Jun 5;18:53. doi: 10.1186/s12866-018-1194-8 (PMC5987490; doi:10.1186/s12866-018-1194-8)
Supplement: Supplementary file 1 — Table S1. Heating method effects on microbial count and main chemical components of tea infusion. Note: All date are presented as mean ± SD, A-Bp < 0.05 in the same column, ND: not detectable, TPs is the abbreviation of tea polyphenols. (DOCX 15 kb) [file 12866_2018_1194_MOESM1_ESM.docx]

Additional file 1:Table S1

Heating method effects on microbial count and main chemical components of tea infusion.

| Heat treatment | Total plate (CFU/mL) | Yeasts and molds (CFU/mL) | Caffeine (mg/L) | TPs (g/L) | Theabrownins (mg/L) |
| --- | --- | --- | --- | --- | --- |
| Control ^c^ | 453±24 | 25±3 | 1227±33.3^A^ | 11.8±0.2^B^ | 687±66.7^A^ |
| 121℃,5min | ND | ND | 1220±13.3^A^ | 11.9±0.3^B^ | 697±36.7^A^ |
| 65℃,30min | 52±46 | 32±1 | 1217±16.7^A^ | 11.5±0.2^B^ | 713±36.5^A^ |
| 75℃,30min | 14±2 | 3±1 | 1223±13.3^A^ | 11.7±0.2^B^ | 746±46.3^A^ |
| 80℃,30min | ND | ND | 1220±23.3^A^ | 11.2±0.2^B^ | 743±50.0^A^ |
| Microwave | 3±1 | 2±0.5 | 1240±20.0^A^ | 10.0±0.5^A^ | 96.±43.3^B^ |

Note: All date are presented as mean ± SD, ^A-B^*p*<0.05 in the same column, ND: not detectable, TPs is the abbreviation of tea polyphenols.
