# Supplementary material for: Biodegradation of caffeine by whole cells of tea-derived fungi Aspergillus sydowii, Aspergillus niger and optimization for caffeine degradation
Source: BMC Microbiol. 2018 Jun 5;18:53. doi: 10.1186/s12866-018-1194-8 (PMC5987490; doi:10.1186/s12866-018-1194-8)
Supplement: Supplementary file 2 — Figure S1. Colony characteristics of strain No. 5 on culture medium. Figure S2. Conidia structure of strain No.5 under optical microscope. Figure S3. Colony characteristics of strain No.1 on culture medium. Figure S4. Conidia structure of strain No.1 under optical microscope. (DOCX 3679 kb) [file 12866_2018_1194_MOESM2_ESM.docx]

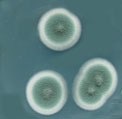

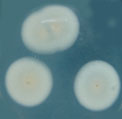


A :Front on PDA B: back on PDA


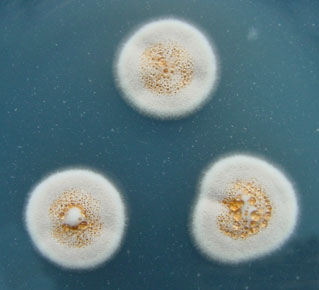

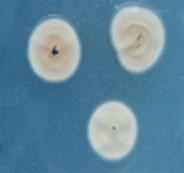


C: front on Czapek`s D: back on Czapek`s

Additional file 2: Figure S1


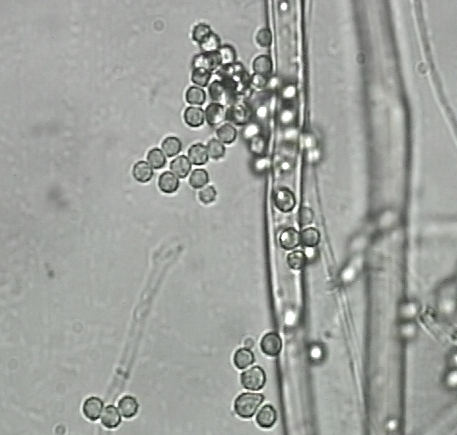

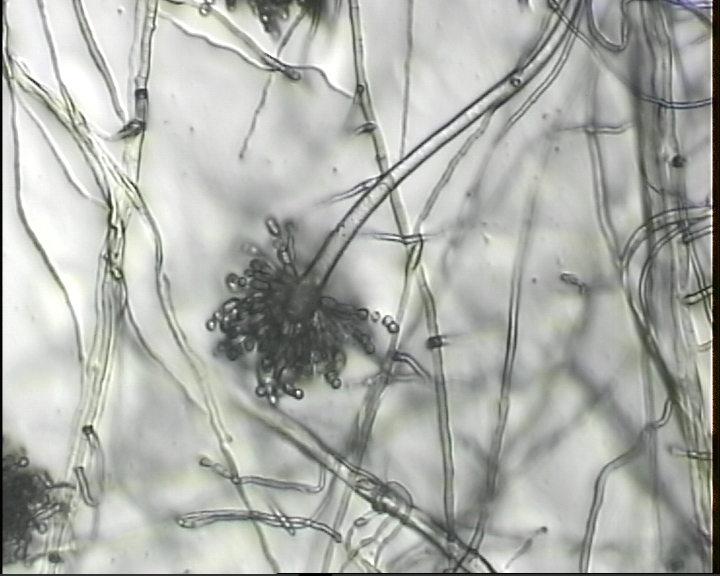


A: conidia (×400) B: conidial head(×200)


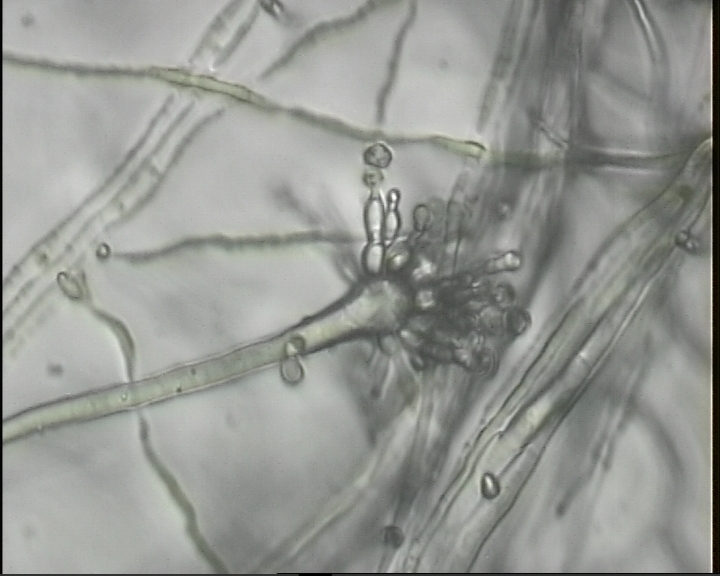

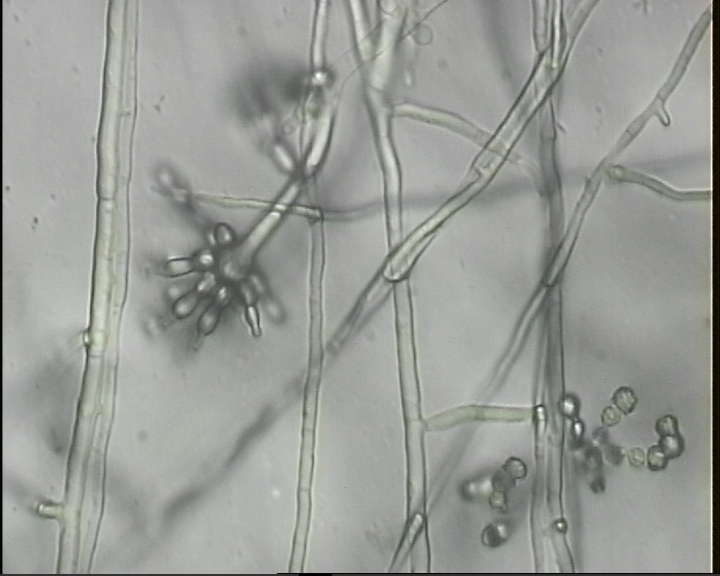


C: double spore structure: metulae, phialides(×200) D: conidiophores (×200)

Additional file 2: Figure S2.


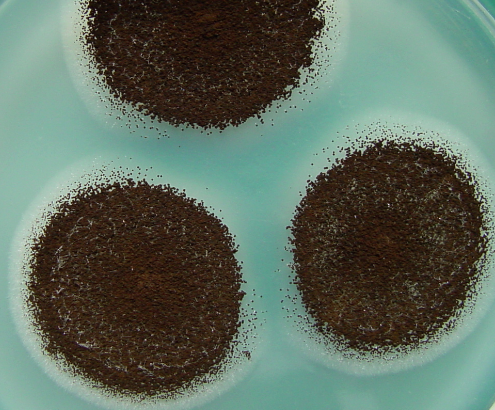

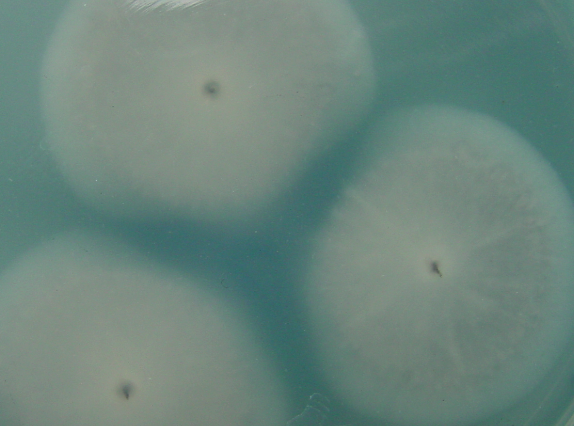


A :Front on PDA B: back on PDA


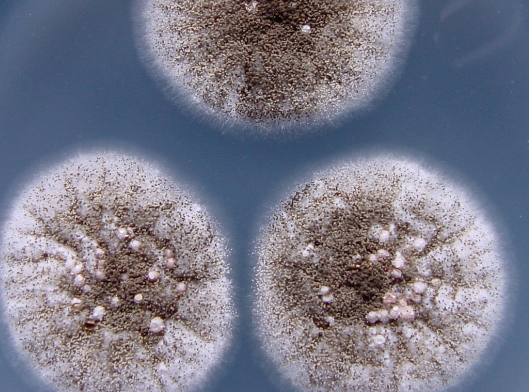

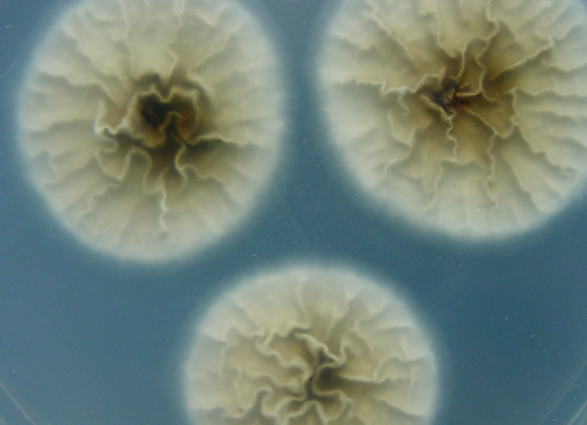


C: front on Czapek`s D: back on Czapek`s

Additional file 2: Figure S3.


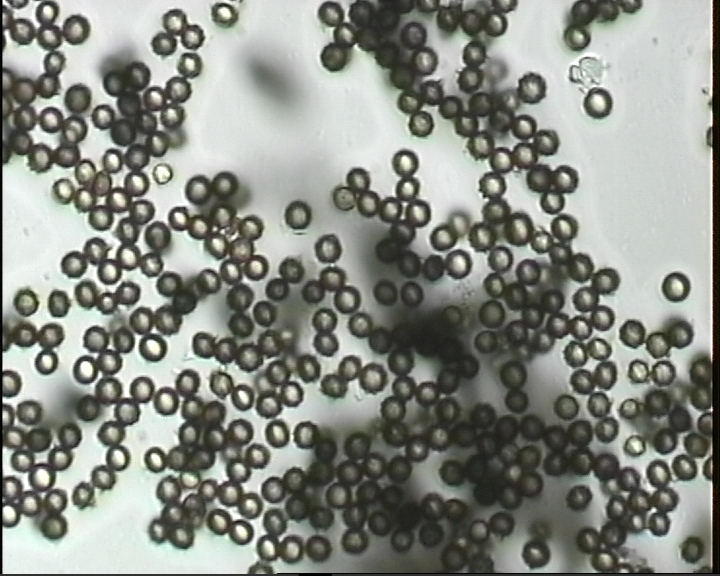


A: conidia (×400)


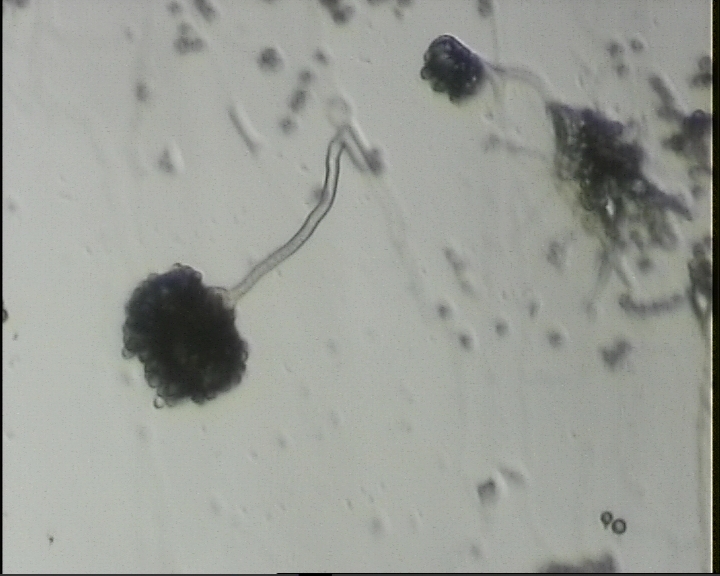

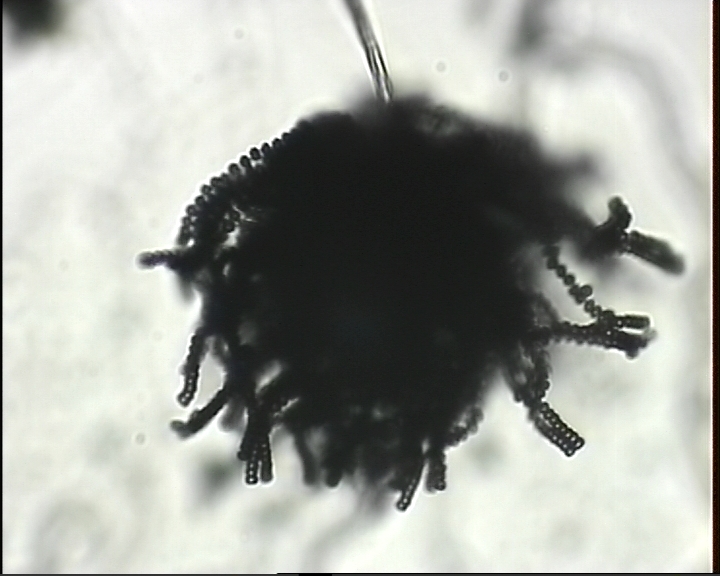


B: condiophore and foot cell (×100) C: chrysanthemum-like conidial head (×200)

Additional file 2: Figure S4.
