# Supplementary material for: Biodegradation of caffeine by whole cells of tea-derived fungi Aspergillus sydowii, Aspergillus niger and optimization for caffeine degradation
Source: BMC Microbiol. 2018 Jun 5;18:53. doi: 10.1186/s12866-018-1194-8 (PMC5987490; doi:10.1186/s12866-018-1194-8)
Supplement: Supplementary file 3 — Figure S5. ITS sequences data of the target strains. (DOCX 16 kb) [file 12866_2018_1194_MOESM3_ESM.docx]

ITS Sequence of strain No. 5 (516bp):

1 CTGCCTCCGGGCGCCCACCTCCCACCCGTGAATACCTAACACTGTTGCTT 50

51 CGGCGGGGAACCCCCTCGGGGGCGAGCCGCCGGGGACTACTGAACTTCA 100

101 TGCCTGAGAGTGATGCAGTCTGAGTCTGAATATAAAATCAGTCAAAACTTT 150

151 CAACAATGGATCTCTTGGTTCCGGCATCGATGAAGAACGCAGCGAACTGC 200

201 GATAAGTAATGTGAATTGCAGAATTCAGTGAATCATCGAGTCTTTGAACG 250

251 CACATTGCGCCCCCTGGCATTCCGGGGGGCATGCCTGTCCGAGCGTCATT 300

301 GCTGCCCATCAAGCCCGGCTTGTGTGTTGGGTCGTCGTCCCCCCCGGGGG 350

351 ACGGGCCCGAAAGGCAGCGGCGGCACCGTGTCCGGTCCTCGAGCGTATGG 400

401 GGCTTTGTCACCCGCTCGACTAGGGCCGGCCGGGCGCCAGCCGACGTCTC 450

451 CAACCATTTTTCTTCAGGTTGACCTCGGATCAGGTAGGGATACCCGCTGA 500

501 ACTTAAGCATATCAAA 516

ITS Sequence of strain No. 1 (546bp):

1 TCTTTGGGCCCACCTCCCATCCGTGTCTATTATACCCTGTTGCTTCGGCG 50

51 GGCCCGCCGCTTGTCGGCCGCCGGGGGGGCGCCTTTGCCCCCCGGGCCCG 100

101 TGCCCGCCGGAGACCCCAACACGAACACTGTCTGAAAGCGTGCAGTCTGA 150

151 GTTGATTGAATGCAATCAGTTAAAACTTTCAACAATGGATCTCTTGGTTC 200

201 CGGCATCGATGAAGAACGCAGCGAAATGCGATAACTAATGTGAATTGCAG 250

251 AATTCAGTGAATCATCGAGTCTTTGAACGCACATTGCGCCCCCTGGTATT 300

301 CCGGGGGGCATGCCTGTCCGAGCGTCATTGCTGCCCTCAAGCCCGGCTTG 350

351 TGTGTTGGGTCGCCGTCCCCCTCTCCGGGGGGACGGGCCCGAAAGGCAGC 400

401 GGCGGCACCGCGTCCGATCCTCGAGCGTATGGGGCTTTGTCACATGCTCT 450

451 GTAGGATTGGCCGGCGCCTGCCGACGTTTTCCAACCATTTTTTCCAGGTT 500

501 GACCTCGGATCAGGTAGGGATACCCGCTGAACTTAAGCATATCAAA 546

Additional File 3:Figure S5
